# Supplementary material for: Identification of photoperiod-induced specific miRNAs in the adrenal glands of Sunite sheep (Ovis aries)
Source: Front Vet Sci. 2022 Jul 22;9:888207. doi: 10.3389/fvets.2022.888207 (PMC9354845; doi:10.3389/fvets.2022.888207)
Supplement: Supplementary file 1 [file Data_Sheet_1.ZIP › Supplementary file/miRDeep2 file.zip!/miRDeep2 file/Know_Structure/SPLP42A1/oar-mir-668.pdf]

miRBase precursor : oar-mir-668
Total read count : 31
oar-miR-668-5p read count : 3
oar-miR-668-3p read count : 24
remaining reads : 4

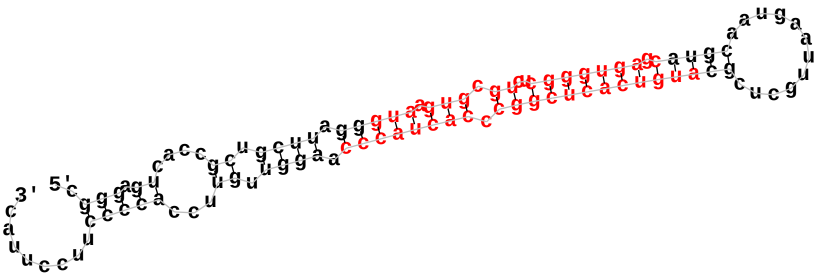

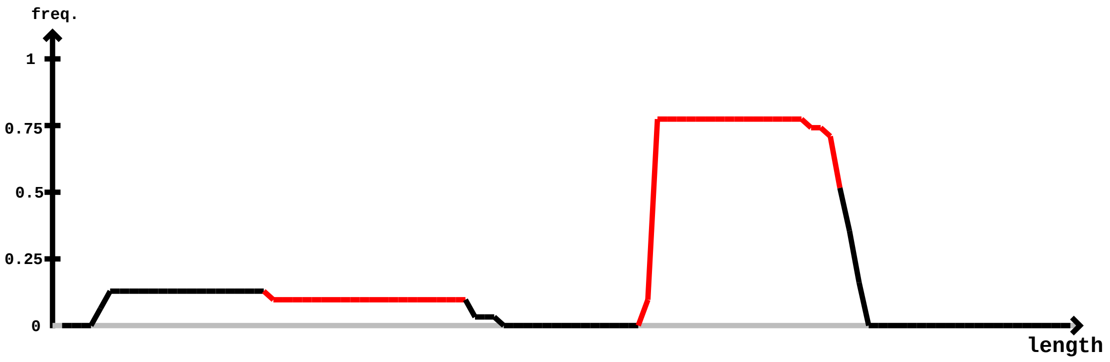


```
                                                                      oar-miR-668-3p
                        oar-miR-668-5p
5'- cgggagucaccgcugcuuaggguaagugcgucucgggugagcaugcaaugaauugcucgcaugucacucggcccacuacccaagguuguuccaccccuuccuuac   -3'    exp
    .(((.((....((.((((.(((((.(((.((..(((((((.(((((............))))))))))))))).)))))))).)))).))...))))).........   reads   mm   sample
    ...gagucaccgcugcuuagg....................................................................................   2       0    seq
    ....agucaccgcugcuuagg....................................................................................   2       0    seq
    .....................guaagugcgucucgggugagc...............................................................   2       0    seq
    .....................guaagugcgucucgggugagcaug............................................................   1       0    seq
    ............................................................augucacucggcccacu............................   1       0    seq
    ............................................................augucacucggcccacuacc.........................   1       0    seq
    ............................................................augucacucggcccacuaccc........................   1       0    seq
    .............................................................ugucacucggcccacuac..........................   1       0    seq
    .............................................................ugucacucggcccacuacc.........................   5       0    seq
    .............................................................ugucacucggcccacuaccc........................   4       0    seq
    .............................................................ugucacucggcccacuaccca.......................   6       0    seq
    .............................................................ugucacucggcccacuacccaa......................   5       0    seq
```
